# Influence of Cooking Methods on Antioxidant Activities of Selected Leafy Vegetables *Gymnema lactiferum*, *Wattakaka volubilis*, and *Argyreia populifolia* in Sri Lanka

**DOI:** 10.1155/2021/6660308

**Published:** 2021-06-01

**Authors:** Sathsara T. Deyalage, Indira Wickramasinghe, Nimesha Amarasinghe, Gayan Thilakarathna

**Affiliations:** ^1^Department of Food Science and Technology, Faculty of Applied Sciences, University of Sri Jayewardenepura, Gangodawila, Nugegoda, Sri Lanka; ^2^National Institute of Post-Harvest Management, Jayanthi Mawatha, Anuradhapura, Sri Lanka; ^3^Department of Animal and Food Sciences, Faculty of Agriculture, Rajarata University of Sri Lanka, Anuradhapura, Sri Lanka

## Abstract

Green leafy vegetables (GLVs) are abundant in bioactive compounds and constitute a crucial part of a balanced diet. Sri Lankan green leafy vegetables which are edible and available for consumption have not been thoroughly investigated, whilst their consumption can deflate the risk of arising several degenerative diseases, such as cancer and cardiovascular diseases. The present study was aimed at evaluating the antioxidant capacities of the leafy vegetables, *Gymnema lactiferum* (Kurignan), *Wattakaka volubilis* (Aguna), and *Argyreia populifolia* (Girithilla), with different thermal treatments (70°C, 120°C, and 170°C) which are used in domestic cooking processes. Heat treatments significantly affect the antioxidant capacity and polyphenolic content of most vegetables analyzed, either increasing or decreasing the concentration of these compounds. In the presence of thermal treatments, *Gymnema lactiferum* (14.52-20.28 mg GAE/g DW) and *Wattakaka volubilis* (19.75-27.13 mg GAE/g DW) showed a significantly higher (*p* < 0.05) total phenolic content. The temperature treatment did not alter the ABTS radical scavenging activity in *Gymnema lactiferum*. In contrast, an increment of ferric reducing antioxidant power (41.26-54.85 mg TE/g DW) and DPPH radical scavenging activity (0.11-0.26 mg TE/g DW) was observed. *Wattakaka volubilis* treated at 170°C appeared to have a significantly higher amount (104.93 ± 1.41 mg TE/g DW) of ferric reducing antioxidant power compared to its raw form. All cooking processes with their corresponding thermal treatments caused a significantly lower (*p* < 0) amount of antioxidant capacity in *Argyreia populifolia.*

## 1. Introduction

Plant-based foods typically contain natural antioxidants that can scavenge free radicals [1]. The key active antioxidant compounds in green leafy vegetables are flavonoids, isoflavones, flavones, catechins, isocatechins, and lignans [2]. Reactive oxygen species (ROS), including free radicals such as singlet oxygen, superoxide anion, hydroxyl radical, and hydrogen peroxide (H_2_O_2_), are continuously produced in the human body during cellular metabolism leading to chronic diseases such as cancer, arthritis, atherosclerosis, Alzheimer's disease, and diabetes [3, 4]. As a result, ample attention focused on the use of antioxidants, especially natural antioxidants to inhibit and prevent damage caused by free radicals and ROS [3]. Despite synthetic antioxidants available in the market owing to the carcinogenic effects, antioxidants of natural origin have drawn significant attention from the society [5].

During the consumption of plant-derived foods, some degree of cooking or processing is performed which enhances their edibility and palatability. These food processing methods can have many effects, not all of which lead to a loss of quality and health properties [6]. Bioavailability and concentration of bioactive compounds in vegetables can be influenced by cooking; this is also involved with inducing significant changes in chemical composition and physical characteristics [3]. On that account, the total phenolic content and antioxidant activities on the basis of their raw form are predicted to be inaccurate [7].

In the Sri Lankan context, GLVs (green leafy vegetables) play a significant role in the diet and people consume GLVs as a part of the main meal with different preparations. These food preparation methods at the household level mainly depend on the taste preferences and convenience rather than their nutrient losses. However, very little information is available in the literature regarding the effects of different cooking methods on the antioxidant activity and the total phenolic contents (TPC) of Sri Lankan leafy vegetables. Therefore, this study was undertaken to investigate and quantify the variation of total phenolic content and the antioxidant activity of selected leafy vegetables: *Gymnema lactiferum*, *Dreaga volubilis*, and *Argyreia populifolia* in Sri Lanka upon different domestic cooking methods. It is vital to determine the available percentage of bioactive compounds that are lost or retained within the GLVs during domestic cooking methods and also to identify the best methods that enhance or reduce degradation of the bioactive compounds.

## 2. Materials and Methodology

### 2.1. Chemicals

All chemicals and solvents used were of analytical grade. Folin-Ciocalteu reagent, gallic acid, 2,2-diphenyl-1-picrylhydrazyl (DPPH), aluminum chloride, sodium acetate, 2,2'-azinobis-(3-ethylbenzo-thiazoline-6-sulfonic acid) diammonium salt (ABTS), 2,4,6-tripyridyl-S-triazine (TPTZ), iron III chloride, quercetin, 6-hydroxy-2,5,7,8-tetramethylchroman-2-carboxylic acid (Trolox), sodium carbonate, and methanol were obtained from Sigma Aldrich, St. Louis, MO, USA, through Analytical Instrument (Pvt) Ltd., Colombo, Sri Lanka.

### 2.2. Leaf Sample Collection and Identification

Fresh, disease-free leaves of *Argyreia populifolia*, *Wattakaka volubilis*, and *Gymnema lactiferum* were collected from Colombo district, Sri Lanka. Three samples (500 g) of each leafy vegetable were collected from three different locations. These collected leafy vegetables were immediately carried to the laboratory in polythene bags. The obtained plant specimens were further identified at the Bandaranaike Memorial Ayurvedic Research Institute, Nawinna, Maharagama. The voucher specimens of the plant samples *Argyreia populifolia* (2048), *Wattakaka volubilis* (2049), and *Gymnema lactiferum* (2041a) have been deposited in the herbarium of the department.

### 2.3. Sample Preparation

The collected three samples of each leafy vegetable (500 g each) were mixed in order to obtain a homogenous sample. Then, edible portions of the leafy vegetables were removed from the thick stalks, and the leafy parts were rinsed in tap water and dried on a paper towel. The cleaned and washed leaves were divided into five equal portions (about 300 g for each cooking treatment). In the beginning, one portion of leafy vegetables (300 g) was stored at -18°C in a zip lock bag as a reference sample. One portion (subdivided into three replicates of 100 g each) was kept as control without any treatment, and the rest were cooked in triplicate (100 g each) in three different methods: tempering (70°C), shallow frying (120°C), and deep-frying (170°C) [8]. The three cooking methods selected are commonly used in Sri Lankan cuisines according to Sri Lankan dietary habits. The samples were cut into small pieces, and each portion was randomly assigned into one of the three treatments (tempering (70°C), shallow frying (120°C), and deep-frying (170°C)) and cooked for 5 minutes. The treated samples were oven-dried at 45°C (model: Leader, UK) until 5% constant moisture content and then coarsely powdered using a mechanical blender to produce fine homogenous samples. This process was repeated for each of three leafy vegetables. The powdered plant materials were stored in sealed triple laminated bags at 4°C until the extraction.

### 2.4. Determination of Proximate Composition of Leafy Vegetables

Standard methods approved by the Association of Official Analytical Chemists [9] were employed to determine the moisture content, crude protein content, fat content, and ash content of oven-dried samples. The moisture content was determined by oven drying the sample to a constant weight at 105°C. The crude protein was determined using the Kjeldahl method (Digester—VELP Scientifica™, DK 6, Italy and Distillation unit—VELP Scientifica™, UDK 129, Italy), and the ash content was assessed using the gravimetric method by muffle furnace (Wisetherm). Fat content was quantified by the Soxhlet method. The total sugar content was determined as per the modified method of Dubois et al. [10].

### 2.5. Sample Extraction for Phytochemical Analysis

The extraction was carried out immediately after cooking treatments following the method reported by Gunathilake and Ranaweera [11] with slight modifications. Approximately 1.25 g of powder sample was extracted with 25 mL of 70% methanol using a mechanical shaker (model: Flash shaker-SF1 Stuart, S/S-R00102429 (150 rpm) for overnight at room temperature). The extract was centrifuged (HERMLE, Z326K) for 15 min at 3000 rpm and filtered through Whatman filter paper (No: 01) to obtain the particle-free extract. The extract volumes were adjusted to 25 mL with 70% (*v*/*v*) methanol before they were stored in the dark at 4°C for further analysis.

### 2.6. Determination of Phytochemicals

#### 2.6.1. Total Phenolic Content (TPC)

The total phenolic content of plant extracts was estimated according to the ISO 14502-1 [12] with some modifications. An aliquot of 0.5 mL of plant extract was mixed with 2.5 mL of 10% (*v*/*v*) Folin-Ciocalteu reagent, and content was mixed thoroughly. Within 3-8 minutes, 2 mL of 7.5% (*w*/*v*) sodium carbonate was transferred to the individual tubes and vortexed well. The final reaction mixture was incubated for 60 minutes at room temperature, and the intensity of the blue-colored complex was measured at 765 nm using a UV-Vis spectrophotometer (model: HATCH DR 600). The control sample consisted of 0.5 mL of 70% (*v*/*v*) methanol with 2.5 mL of Folin-Ciocalteu reagent and 2 mL of sodium carbonate solution. The experiment was triplicated for each sample. Total phenolic content was determined as Gallic Acid Equivalent (GAE) by a calibration curve (*r*^2^ = 0.9973) plotted using gallic acid reference standard at concentrations of 10, 20, 30, 40, 50, 60, and 70mg/L. The results were expressed as milligrams of gallic acid equivalent (GAE) per g of plant material (DW).

#### 2.6.2. Total Flavonoid Content (TFC)

Total flavonoid content was determined by the method suggested by Ramkissoon et al. [13]. Briefly, 2.50 mL aliquot of plant extract, 150 *μ*L of 5% sodium nitrite (*w*/*v*), 150 *μ*L of 10% aluminum chloride (*w*/*v*), and 1 mL of 1 M sodium hydroxide were pipetted into tubes, respectively. After that, the solution volume was increased up to 5 mL by adding distilled water. The content of the mixture was vortexed properly, and optical density was measured at 510 nm using a UV/VIS Spectrophotometer. The experiment was triplicated for each sample. Total flavonoid content was determined by a calibration curve (*r*^2^ = 0.9915) plotted using quercetin reference standard at concentrations of 1000, 500, 250, 125, 62.5, and 31.25 mg/L. The results were expressed as milligrams of quercetin equivalent (QEs) per g of plant material (DW).

#### 2.6.3. ABTS Assay

ABTS radical scavenging activity was determined according to the method employed by Proestos et al. [14] with slight modifications. ABTS reagent solution was freshly prepared by mixing 7 mmol of ABTS solution treated with 2.45 mmol of potassium persulfate. ABTS powder and potassium persulfate powder were individually dissolved in deionized water to the required concentration and then were mixed in a 1 : 1 ratio in a centrifuge tube. After 16 hours of incubation in the dark at 37°C, the resultant dark blue color ABTS solution was diluted with 70% (*v*/*v*) methanol in a 1 : 40 ratio until the absorbance reading reached 0.800 ± 0.005 at 734 nm. Plant extracts of 0.04 mL were mixed with 4 mL of ABTS/K_2_S_2_O_8_ stock solution. The reaction mixture was vortexed well for proper mixing and kept in the dark for 15 minutes. The absorbance was measured at 734 nm using UV-Vis spectrophotometer. The experiment was triplicated for each sample. The antioxidant capacity was determined by a calibration curve (*r*^2^ = 0.9947) plotted using the Trolox reference standard at concentrations of 400, 300, 200, 100, 50, and 25 mg/L. The results were expressed as millimoles of Trolox equivalent (TE) per g of plant material (DW).

#### 2.6.4. Ferric Reducing Antioxidant Power Assay (FRAP Assay)

Ferric reducing antioxidant power levels were determined using a method proposed by Benzie and Strain [15] with minor modifications. The working FRAP reagent was prepared by mixing 10 volumes of 300 mM acetate buffer (pH 3.6), with 1 volume of 10 mM TPTZ (2,4,6-tripyridyl-S-triazine) in 40 mM HCl and with 1 volume of 20 mM ferric chloride. All the required solutions were freshly prepared before the analysis. 150 *μ*L of samples were mixed with 4.5 mL of prepared FRAP reagent, and the reaction mixture was incubated for 30 minutes at 37°C. The absorbance of the samples was measured at 593 nm using UV-Vis spectrophotometer. The experiment was triplicated for each sample. The antioxidant capacity was as quantified by a calibration curve (*r*^2^ 132 = 0.9921) plotted using the Trolox reference standard at concentrations of 100, 80, 70, 60, 50, 40, 30, and 20 mg/L. The results were expressed as milligrams of Trolox equivalent (TE) per g of plant material (DW).

#### 2.6.5. 2,2-Diphenyl-1-Picrylhydrazyl Assay (DPPH Assay)

DPPH radical scavenging activity was assessed by using the procedure suggested by Petlevski et al. [16] with some modifications. Briefly, 2.0 mL of 0.16 mM DPPH methanolic solution was added to 2.0 mL of aqueous methanolic plant extract (sample). The mixture was vortexed for 1 min and then left to stand at room temperature in the dark. After, 30 min absorbance was read at 517 nm using UV-Vis spectrophotometer. The experiment was triplicated for each sample. The antioxidant capacity was quantified by a calibration curve (*r*^2^ = 0.9946) plotted using the Trolox reference standard at concentrations of 25, 12.5, 6.25, 3.125, and 1.5625 mg/L. The results were expressed as milligrams of Trolox equivalent (TE) per g of plant material (DW).

### 2.7. Statistical Analysis

Each parameter was analyzed in triplicate. Data were recorded as the mean ± standard deviation (SD). One-way analysis of variance (ANOVA) was applied for each parameter. Tukey test was applied for the mean comparison 95% significance level. Balance ANOVA was applied to study the effect of two variables: heat treatment and variety. All statistical analysis was performed using the MINITAB 17 software package.

## 3. Results and Discussion

### 3.1. Proximate Composition of Leafy Vegetables

Standard procedures were followed to determine the moisture, crude protein, fat, and ash of three leafy vegetables, and their proximate composition is presented in Table 1.

The values obtained for the proximate composition are consistent with the previous data available in the literature. The moisture content of the samples ranged between 76.54 and 83.23%. These values of the moisture content were corroborated with the results (70-90%) reported by Singh et al. [17]. Mean differences of the three varieties revealed no significant differences across *Wattakaka volubilis* and *Argyreia populifolia* under 0.05 significance level. The relatively high moisture content depicts that these leafy vegetables need care for appropriate preservation as they would be prone to deterioration since it may induce a greater activity of water-soluble enzymes and coenzymes involved in the metabolic activities of these leafy vegetables [18].

Ash content was relatively high with values ranging from 12.16 ± 0.04% for *Argyreia populifolia* to 13.08 ± 0.11% for *Wattakaka volubilis*. The high ash content of the *Wattakaka volubilis* depicts the presence of high mineral content since the high ash content is a reflection of the level of inorganic elements such as calcium, zinc, magnesium, copper, and potassium in the vegetable. These minerals act as inorganic cofactors in metabolic processes [19]. According to the mean differences of ash content in the three varieties, no significant differences were observed across *Wattakaka volubilis* and *Gymnema lactiferum* under 0.05 significance level. The results of the present study concurred well with the values obtained for *Centella asiatica* (13.26 ± 1.60%) by Singh et al. [17]. Nonetheless, the ash values were slightly lower in contrast to the values reported by [18] for *A. hybridus* leaves (17.70 ± 0.01%).

The protein content of *Wattakaka volubilis* (19.82 ± 0.43%) (DW) was reported as the highest where the least value was reported from the *Argyreia populifolia* (16.68 ± 0.26%) (DW). As per the statistical analysis, a significant difference (*p* < 0.05) was observed in the crude protein values for the three leafy vegetables. These values were quite lower compared to those of *Moringa olieofera* (27.51 ± 0.00%) as reported by Oduro et el. [19]. The mean lipid content showed no significant difference in three leafy vegetables tested at the 0.05 significance level, although *Wattakaka volubilis* had the highest lipid content (5.25 ± 0.07%) (DW) among them. The fat content reported in the study was moderate when compared to the data of the previous literature [20] which was conducted for leafy vegetables.

*Argyreia populifolia* (51.17 ± 0.60%) (DW) stands out to be a better source (*p* < 0.05) of carbohydrate than other leafy vegetables tested in the present study following *Gymnema lactiferum* and *Wattakaka volubilis.* Conferring to the statistical analysis, the mean values of all three leafy vegetables were significantly different (*p* < 0.05) in carbohydrate content. The results of the present study tallied with the literature of [12, 20]. On the contrary, obtained values for carbohydrate content were quite higher compared to the dry basis values reported for the *Amaranthus hybridus* and *Curcubita pepo*, respectively, 14.36 ± 0.01% and 12.94 ± 0.01% [18].

### 3.2. Determination of Phytochemical Analysis

#### 3.2.1. Total Phenolic Content (TPC)

Results of the total phenolic content of three leafy vegetables subjected to different thermal treatments are presented in Table 2.

According to the statistical analysis, it was observed that there is a significant difference in total phenolic content with two factors: variety and temperature (*p* < 0.05). Raw forms of three leafy vegetables were significantly different (*p* < 0.05) in their total phenolic content and increased in the order of *Gymnema lactiferum* (14.52 ± 0.19 *mg* GAE/g DW), *Wattakaka volubilis* (20.92 ± 0.54 *mg* GAE/g DW), and *Argyreia populifolia* (24.48 ± 0.67 *mg* GAE/g DW). The total phenolic content reported for *Gymnema lactiferum* in this study was higher than the reported value in the previous literature (11.03 ± 0.42 GAE/g DW) [11]. Relevant to the observed values, earlier findings of leafy vegetables represent relatively lower values for *Amaranthus* species (3.4-4.5 mg GAE/g FW) and *Alternanthera sessilis* green (3.70 ± 0.21 GAE/g FW) [21, 22].

When considering the heat effect, the highest total phenolic content was reported from the 170°C treated *Wattakaka volubilis* with a mean of 27.13 ± 0.05 mg GAE/g DW. The least value was observed from the 70°C treated sample of the *Argyreia populifolia* with a mean of 10.24 ± 0.22 mg GAE/g DW. *Argyreia populifolia* leaves nearly exhibited more than two times lower phenolic content at 170°C treated samples, concerning the initial form. Similarly, after 70°C treatment, total phenolic content was significantly decreased (*p* < 0.05) by 58% concerning their original content. There was no significant difference among the loss of phenolic content at 70°C and 120°C treated samples of this variety. This reduction can be caused by the breaking down of polyphenols in these leafy types during cooking. Both varieties of *Gymnema lactiferum* and *Wattakaka volubilis* showed a significantly higher (*p* < 0.05) total polyphenol content in their treated samples compared with their initial samples. *Wattakaka volubilis* and *Gymnema lactiferum* revealed an increasing pattern of phenolic content with all treatments. In *Wattakaka volubilis*, both 120°C and 170°C treated samples were not significantly different (*p* > 0.05) and comprised similar amounts of phenolic contents. In this current study, the leaves of *Gymnema lactiferum* showed 140% higher phenolic content at 170°C treated sample than in its original form. As mentioned by Faller and Fialho [23], coriander, mint, and spinach had a significant increase in phenolic content with cooking methods such as conventional, pressure, and microwave, and this increase was ranging from 125–211% has been taken from Sreeramulu [7]. The possible explanation for the increasing trends of phenolic contents during various cooking methods could be that individual phenolics may sometimes increase because heat can break supramolecular structure which might make the phenolic compounds react better with the reagents [24]. Besides, each plant presents different types of phenolic compounds, along with different bonds between these phytochemicals and the cell structures. These variations can lead to higher or lower cleavage of phenolic bonds according to the type of heat applied and the food being analyzed [23]. Moreover, the heat treatments could inactivate polyphenol oxidases, preventing oxidation and polymerization of polyphenols [25].

#### 3.2.2. Total Flavonoid Content (TFC)

The results obtained for the total flavonoid content of the selected leafy vegetables were summarized in Table 3. Total flavonoid content in the raw form of three leafy vegetables ranged from 6.43 ± 0.10 (mg QE/g DW) to 20.01 ± 0.64 (mg QE/g DW). Statistical analysis revealed that the raw form of all three leafy vegetables was significantly different (*p* < 0.05) in total flavonoid content. *Argyreia populifolia* was the species with the highest flavonoid concentration, roughly thrice the value found in *Wattakaka volubilis*. Among three GLVs analyzed, total flavonoid content in *Wattakaka volubilis* and *Gymnema lactiferum* was in accordance with the values reported for parsley (14.35 mg QE/g DW) and red kale (6.57 mg QE/g DW) in previous literature [5]. The observed value of *Argyreia populifolia* was well corroborated with the reported value for *Alternanthera sessilis* (21.51 ± 0.46 mg QE/g DW) [26].

Temperature treatment was not significantly altering the flavonoid content of the 70°C and 120°C treated samples of *Wattakaka volubilis*. In contrast, the total flavonoid content in *Wattakaka volubilis* had relatively lower values than the initial sample. Unexpectedly 70°C treated samples of all three varieties showed significantly lower (*p* < 0.05) amounts compared to other treatments carried out in the study. Both A*rgyreia populifolia* and *Gymnema lactiferum* exhibited the same fluctuation of flavonoid content with the temperature ranging from 70°C–170°C. The percentage decreases of flavonoid content in 170°C treated samples of *Wattakaka volubilis and Argyreia populifolia* were 24% and 32%, respectively. However, the content of flavonoids found in 170°C treated *Gymnema lactiferum* was twice more than that in the untreated sample. As stated in previous literature, flavanols represent a different behavior depending on the type of cooking performed. Baking and sautéing lead to a 7–25% gain in quercetin concentration [23]. Accordingly, variation in losses and gains of flavonoids due to cooking treatments in the leafy types studied could be due to the types of heat treatment, the nature of leaves, and the forms of the flavonoids present in the plant matrices.

#### 3.2.3. ABTS Assay

Table 4 presents the ABTS radical scavenging activity of raw and cooked leafy vegetables. Among the uncooked samples of three leafy vegetables, the highest antioxidant capacity was detected for *Argyreia populifolia* with a mean of 4.10 ± 0.05 mM TE/g. The least antioxidant activity was reported from *Gymnema lactiferum* where it was four times lower than that of *Argyreia populifolia*. The results of the current study were not in line with the previous literature [27]. These ABTS radical scavenging activities appeared to have much higher values concerning the values reported for lettuce (85.8 ± 5.34 *μ*mol/g DW) and spinach (41.2 ± 5.24 *μ*mol/g DW).

When considering the treated samples, results showed that ABTS radical scavenging activities were reduced during all treatments in *Gymnema lactiferum* and *Argyreia populifolia* compared with their fresh leaves. The fluctuation of ABTS radical scavenging activity with the thermal treatments evidenced no significant differences among all treated samples in *Gymnema lactiferum* (*p* < 0.05). Similarly, in the *Wattakaka volubilis*, no significant difference was observed except 170°C treated sample (*p* < 0.05). Overall, the antioxidant activities in various green leafy vegetables were affected differently by thermal processing.

#### 3.2.4. Ferric Reducing Antioxidant Power Assay (FRAP Assay)

The results of the evaluation of ferric reducing antioxidant power of leafy vegetables subjected to different heat treatments are presented in Table 5. There was a significant difference in ferric reducing antioxidant power with two factors; variety and temperature (*p* < 0.05). The uncooked samples of three leafy vegetables *Gymnema lactiferum* (41.26 ± 0.87 mg TE/g DW) and *Wattakaka volubilis* (43.36 ± 1.60 mg TE/g DW) exhibited nearly three times decrease in antioxidant capacity following *Argyreia populifolia.* These results of the present study slightly fluctuate with previous reports, showing lower values for antioxidant capacities. This can be mainly due to the different solvents used in these experiments. Aqueous methanolic extracts had greater ferric reducing powers than water extracts, as aqueous methanol extracts demonstrated the highest reducing power among the vegetable samples tested in the previous studies [28]. A decreasing trend in reducing power was noted in *Argyreia populifolia* with the application of thermal treatment. On the contrary, the other two varieties *Gymnema lactiferum* and *Wattakaka volubilis* are having similar patterns of increase in antioxidant activity except for 70°C treated samples. 170°C treated samples in both varieties appeared to have a significant increment of radical scavenging activity by 32% for *Gymnema lactiferum* and 142% for *Wattakaka volubilis*. The value of percent gain in reducing power also indicates the effectiveness of the cooking process on the antioxidant potential of leafy vegetables [3]. Referring to previous investigations, it can be noted that upon different cooking methods, vegetables possessed a significantly higher capacity to reduce free radicals than the raw samples [29]. The findings of the present study were in agreement with the previous literature [30] which observed increased FRAP values of onion varieties with higher temperatures, relative to the onion at ambient temperature.

#### 3.2.5. 2,2-Diphenyl-1-Picrylhydrazyl Assay (DPPH Assay)

The results obtained for the DPPH radical scavenging capacities of the selected leafy vegetables were shown in Table 6. In this process, by offering hydrogen atoms or by electron donation, polyphenols can quench DPPH radicals and convert them to a colorless substance. Hence, the greater the percentage of inhibition of free radical activity, the more potent the antioxidant capacity of the extract in terms of hydrogen atom-donating capacity [11]. Among three leafy vegetables, the least free radical scavenging activity of 0.11 mg TE/g DW was reported in the row form of *Gymnema lactiferum*. Previous studies on other leafy vegetables have shown relatively lower values for lettuce 27.94 (*μ*g TE/g DW), green *Amaranth* (28.58 *μ*g TE/g DW) [31]. These deviations can be caused by the limitations involved in the DPPH method which can further misjudge the antioxidant capacity: it may react respond slowly or be neutral to many antioxidants; reaction kinetic with antioxidants does not tend to be linear to DPPH concentrations, and reaction of DPPH with certain phenolic structures could not be completed, reaching a state of equilibrium [32].

When considering the DPPH radical scavenging activity of the cooked samples, there was a significant difference in DPPH free radical scavenging activity with two factors: variety and temperature (*p* < 0.05). In general, an increasing trend in DPPH radical scavenging activity on the heat treatment was observed in *Gymnema lactiferum*; values ranged from 0.11 ± 0.00 to 0.26 ± 0.00 mg TE/g DW. Among that, 170°C treated sample of *Gymnema lactiferum* exhibited an enormous increase by 124% compared to the uncooked sample. The previous literature confirms the positive increment of the antioxidant activity. It is stated that heating increased the antioxidant activity of carrot leaves extracts significantly (*p* < 0.05). The enhanced activity might be attributed to the fact that thermal processing can induce the development of compounds with antioxidant properties such as Maillard reaction products or boost the antioxidant activity of naturally occurring antioxidants [33]. In the case of *Wattakaka volubilis*, there was no significant difference between the raw form and the sample treated at 70°C under 0.05 significance level. In contrast, *Argyreia populifolia* was observed with a slight gain of radical scavenging capacity by 6% and 9%, respectively, at 70°C and 120°C. However, at 170°C, radical scavenging activity was lost by 28%. This may be due to losses or degradation of certain types of phenolic compounds or other DPPH free radical-scavenging components during the heat treatments. These results support previous literature [34] which reported that the radical-scavenging activity would decrease if the vegetables were exposed to heat, such as blanching.

## 4. Conclusion

The findings of the present study indicate that the total phenolic content, total flavonoid content, and the antioxidant activities of selected green leafy vegetables are significantly altered during domestic cooking methods such as tempering, shallow frying, and deep-frying. Due to the addition of heat treatments, the content of phenol and antioxidants increased in *Gymnema lactiferum* and *Wattakaka volubilis* compared to their uncooked state. Deep frying at 170°C for 5 minutes is proven to be the best for these two leafy vegetables in terms of preserving antioxidant properties. On the contrary, in *Argyreia populifolia*, the total phenol content and antioxidant content decreased significantly when subjected to cooking treatments. This overview will be positively beneficial to researchers, nutritionists, and consumers to evaluate AOA and articulate antioxidant-rich therapeutic diets and even as commercial antioxidant-rich preparations from plant foods. Further, not only fresh but also treated leafy vegetables can be used as a food additive and also as a raw material for functional food production.

## Figures and Tables

**Table 1:** Proximate composition of selected leafy vegetables.

Variety	Moisture % (WB)	Moisture % (DW)	Ash% (DW)	Protein % (DW)	Fat % (DW)	Carbohydrate % (DW)
Gl	76.54 ± 0.24^b^	7.32 ± 0.17^b^	12.93 ± 0.04^a^	17.99 ± 0.54^b^	4.42 ± 0.48^a^	37.20 ± 0.64^b^
Wv	83.23 ± 0.24^a^	7.76 ± 0.10^a^	13.08 ± 0.11^a^	19.82 ± 0.43^a^	5.25 ± 0.07^a^	24.30 ± 1.63^c^
Ap	83.01 ± 0.46^a^	7.75 ± 0.06^a^	12.16 ± 0.04^b^	16.68 ± 0.26^c^	4.54 ± 0.44^a^	51.17 ± 0.60^a^

Values are presented as mean ± SD, *n* = 3. Values in the same column having the same superscript letters are not significantly different (*p* > 0.05). Scientific names of leafy vegetable have been abbreviated as follows: Gl: *Gymnema lactiferum*; Wv: *Wattakaka volubilis*; Ap: *Argyreia populifolia*.

**Table 2:** Total phenolic content of thermally treated leafy vegetables (DW).

Variety	Processing temperature			
	Control	70°C	120°C	170°C
Gl	14.52 ± 0.19^f^	15.34 ± 0.08^e^	17.23 ± 0.20^d^	20.28 ± 0.12^c^
Wv	19.75 ± 0.08^c^	20.06 ± 0.15^c^	26.90 ± 0.10^a^	27.13 ± 0.05^a^
Ap	24.48 ± 0.67^b^	10.31 ± 0.10^h^	10.24 ± 0.22^h^	12.07 ± 0.14^g^

Values are presented as mean ± SD, *n* = 3. Values having the same superscript letters are not significantly different (*p* > 0.05). Scientific names of the leafy vegetables have been abbreviated as follows: Gl: *Gymnema lactiferum*; Wv: *Wattakaka volubilis*; Ap: *Argyreia populifolia*.

**Table 3:** Total flavonoid content of thermally treated leafy vegetables (DW).

Variety	TFC (mg QE/g)			
	Control	70°C	120°C	170°C
Gl	14.29 ± 0.28^d^	9.55 ± 0.12^f^	22.60 ± 1.01^b^	28.84 ± 0.62^a^
Wv	6.43 ± 0.10^h^	5.40 ± 0.27^hi^	6.05 ± 0.12^hi^	4.89 ± 0.19^i^
Ap	20.01 ± 0.64^c^	8.19 ± 0.46^g^	12.77 ± 0.14^e^	13.52 ± 0.38^de^

Values are presented as mean ± SD, *n* = 3. Values having the same superscript letters are not significantly different (*p* > 0.05). Scientific names of the leafy vegetables have been abbreviated as follows: Gl: *Gymnema lactiferum*; Wv: *Wattakaka volubilis*; Ap: *Argyreia populifolia*.

**Table 4:** ABTS radical scavenging capacity of thermally treated leafy vegetables (DW).

Variety	ABTS (mM TE/g)			
	Control	70°C	120°C	170°C
Gl	1.03 ± 0.13^d^	0.95 ± 0.03^d^	0.78 ± 0.04^d^	0.95 ± 0.02^d^
Wv	3.63 ± 0.06^ab^	3.69 ± 0.35^ab^	3.82 ± 0.21^ab^	4.09 ± 0.07^a^
Ap	4.10 ± 0.05^a^	2.64 ± 0.06^c^	2.34 ± 0.07^c^	3.42 ± 0.37^b^

Values are presented as mean ± SD, *n* = 3. Values having the same superscript letters are not significantly different (*p* > 0.05). Scientific names of the leafy vegetables have been abbreviated as follows: Gl: *Gymnema lactiferum*; Wv: *Wattakaka volubilis*; Ap: *Argyreia populifolia*.

**Table 5:** Ferric reducing antioxidant power of thermally treated leafy vegetables (DW).

Variety	FRAP (mg TE/g)			
	Control	70°C	120°C	170°C
Gl	41.26 ± 0.85^gh^	38.51 ± 0.47^h^	51.22 ± 0.76^f^	54.85 ± 0.47^e^
Wv	43.36 ± 1.60^g^	41.39 ± 0.86^gh^	74.44 ± 0.38^d^	104.93 ± 1.41^c^
Ap	120.34 ± 1.35^a^	115.71 ± 1.77^b^	115.58 ± 0.78^b^	107.69 ± 0.57^c^

Values are presented as mean ± SD, *n* = 3. Values having the same superscript letters are not significantly different (*p* > 0.05). Scientific names of the leafy vegetables have been abbreviated as follows: Gl: *Gymnema lactiferum*; Wv: *Wattakaka volubilis*; Ap: *Argyreia populifolia*.

**Table 6:** DPPH radical scavenging activity of thermally treated leafy vegetables (DW).

Variety	DPPH (mg TE/g)			
	Control	70 °C	120°C	170°C
Gl	0.11 ± 0.00^g^	0.24 ± 0.00^e^	0.23 ± 0.00^de^	0.26 ± 0.00^b^
Wv	0.25 ± 0.01^bc^	0.25 ± 0.00^bc^	0.23 ± 0.00^de^	0.24 ± 0.00^cd^
Ap	0.25 ± 0.00^b^	0.27 ± 0.00^a^	0.28 ± 0.00^a^	0.20 ± 0.00^f^

Values are presented as mean ± SD, *n* = 3. Values having the same superscript letters are not significantly different (*p* > 0.05). Scientific names of the leafy vegetables have been abbreviated as follows: Gl: *Gymnema lactiferum*; Wv: *Wattakaka volubilis*; Ap: *Argyreia populifolia*.

## Data Availability

The data used to support the findings of this study are included within the article.
